# Impact of Inperson Training on Health Care Provider Knowledge of the Symptoms and Treatment of Pediatric Cancer in Mwanza Region

**DOI:** 10.1200/GO.22.61000

**Published:** 2022-05-05

**Authors:** Norbert Mtenga, Glorian Nnko, Goodluck Nchasi, Eric Magese, Shabani Rajabu, Hillary Sued, Heronima Joas, Erica Sanga, Kristin Schroeder

**Affiliations:** ^1^ Catholic University of Health and Allied Science, Mwanza, Tanzania; ^2^ Bugando Medical Centre, Mwanza, Tanzania; ^3^ National Institute of Medical Research—Lake Zone, Dar-es-Salaam, Tanzania; ^4^ Duke University, Durham, NC

## Abstract

In Tanzania, < 20% of the estimated 3,500 children who develop cancer each year present to one of the three cancer treatment centers. Of those who do present, there is an average of 89 days between when they present to the local health care center and their cancer center evaluation. Our aim was to assess the baseline pediatric cancer knowledge among medical providers at health centers and district hospitals in the Mwanza region and evaluate the impact of a training program on cancer knowledge and symptom recognition, and ultimately reduce referral delay.

**METHODS:**

One provider from each of the 69 Mwanza region health care centers and district hospitals was selected by district non communicable disease coordinators to participate in a one day training program. Training education strategies included didactic, small group discussions and interactive sampling. Topics included childhood cancer epidemiology, symptoms recognition, diagnostic evaluation, and referral options. Participants completed re- and post-training surveys to determine knowledge change.

**RESULTS:**

A total of 80% (n = 56) of invited health centers participated in the training. In the pre survey, while all participants knew what cancer was (uncontrolled cellular division), 82% of were not aware that there was potentially curative treatment available, and 35% did not know which hospitals provided cancer treatment in their region. Post training, overall knowledge scores significantly increased (73%-81%, *P* < .001), with a > 20% score change for key content areas including symptom recognition and appropriate referral location hospital.

**CONCLUSION:**

Community medical provider knowledge about childhood cancer is low within the Mwanza Region. In person short training is an effective strategy to increase key knowledge areas that are known to contribute to referral delay. Future research will evaluate the impact of this training on referral rates and referral delay to the regional cancer referral hospital.

